# Contributions to Operative Orthopædia; on the Subcutaneous Division of Contracted Muscles and Their Tendons

**Published:** 1839-10

**Authors:** 


### ART. V.

1. *Beiträge zur operativen Orthopädik, oder Erfahrungen über die subcutane Durchschneidung verkürzter Muskeln und deren Sehnen.* Von Dr. LOUIS STROMEYER, Königl. Hofchirurgus, &c. zu Hannover. *Mit* 8 *Lithographirten Tafeln.—Hannover,* 1838. 8vo, pp. 154.

*Contributions to Operative Orthopœdia; or, Practical Observations on the Subcutaneous Division of Contracted Muscles and their Tendons.* By Dr. LOUIS STROMEYER, of Hanover. *Illustrated by* 8 *Lithographic Plates.—Hanover,* 1838.

2. *Handbuch der Plastischen Chirurgie.* Von Dr. EDUARD ZEIS, Doctor der Medicin und Chirurgie, &c. (19 *Abtheilung.*) — *Berlin,* 1838. 8vo.

*Manual of Plastic Surgery.* By Dr. EDWARD ZEIS, of Dresden. (*Chapter XIX. on the Division of Tendons.*)—*Berlin,* 1838.

3. *Mémoire sur la Section du Tendon d'Achille dans le Traitement des Pieds-bots.* Par M. BOUVIER. *Agrégé libre à la Faculté de Médecine à Paris, &c.—Paris,* 1838. 4to, pp. 73.

*Memoir on the Section of the Tendo-Achillis in the treatment of Club-foot.* By M. Bouvier, of Paris. *Forming part of Vol. VII. of the Memoirs of the Academy of Medicine.—Paris*, 1838.

4. *A Treatise on the Nature of Club-foot, and analogous Distortions; including their Treatment, both with and without Surgical Operation. Illustrated by a series of Cases and numerous Practical Instructions.* By W. J. Little, m.d., Lecturer on Comparative Anatomy at the Medical School of the London Hospital, &c.—*London*, 1839. 8vo, pp. 276.

At the close of an article on "Plastic Surgery," in our Fourteenth Number, we promised to pursue our notice of Dr. Zeis's work, by passing in review that division of it which treats of the operations for the cure of deformities resulting from contracted muscle or tendon; and having now added, as text-books, the other works which head the present paper, we proceed to redeem our pledge. Although the operations alluded to by no means form an essential part of plastic surgery, yet we cannot but coincide with Dr. Zeis in regarding the two subjects as legitimately allied. The object of both is rather the reparation of some existing deformity than the cure of a disease; and this consideration, taken in connexion with the fact of their having sprung almost simultaneously into general notice, points to the discussion of the one as an appropriate sequel to that of the other.

There are few more interesting sources of meditation than that which is afforded by a comparison between the state of our past and present knowledge, as we review the progressive advance of the human mind through ages; and we have slight sympathy with the chilling and mis-named philosophy which can regard with indifference the mighty achievements of the intellect of man, unless moved by the selfish promptings of the economist and calculator. Yet how difficult is it for us to place a due value upon discoveries with which we have been always familiar: how little can we appreciate the obstacles presented by previous ignorance, limited means of observation or experiment, and, worse than all, by the confirmed prejudice of long-established error! Indeed, a certain proportion of wonder at the obtuseness of the discoverer's predecessors, appears to be almost inseparable from a contemplation of the simplicity which a newly-developed law presents. Thus, for example, we are far more prone to wonder how it was possible for any one conversant with the simple anatomy of the circulating system to mistake the real use of its various component parts, than to admire the sagacity of Harvey, which led him, undisturbed by the many difficulties which encompassed him, to an exposition of the real and appropriate functions of the heart, the arteries, and the veins; and thus, now that the noseless, the wry-necked, and the lame are restored to happiness and society, we cannot forbear reflecting on the singularity of the fact that we should have so long been content, whilst cultivating the maiming department of our art to the utmost, to allow this beautifying and comfort-restoring division to have remained comparatively neglected.

The operations which we are about to discuss are available for the cure of certain deformities of the neck and extremities alone ; and of these, such only as have for their cause the contraction or shortening of some portion of the active locomotive organs : it becomes the operator, therefore, to make the state of the articulation, which forms the angle of contraction, his peculiar study, before he attempts to remedy that which original malformation or disease of the hard parts may have rendered irremediable. Thus it is the abnormal condition of the spinal column which, in nearly every instance, is the source of the various distortions of the trunk, and which necessarily precludes the possibility of relief from section of muscle or tendon. In most of these cases the alteration in the relation of the bones to one another is the primary condition; and the resulting wasting or shrinking of the muscles is altogether secondary or consequent; whereas precisely the converse is the case in curable instances of contracted neck or extremities. There are, doubtless, exceptions to the former of these positions, in which much may be done by attention to the proper employment and development of the muscles; and to such of our readers as may take an interest in the subject, we recommend the perusal of Dr. Stromeyer's little work, "Ueber Paralyse der Inspirations-Muskeln," as well as a paper by Dr. Gunther, of Hamburg, which will be found in our Fourth Volume, p. 509. Further, the contraction of articulations consequent on destruction of the superficial soft parts is a condition to which both the neck and extremities are obnoxious; but these, for the most part, incurable deformities require an essentially different treatment, and do not form a part of our present subject; we shall, therefore, with trifling exceptions, confine ourselves, on the present occasion, to the discussion of such cases only as find their remedy in section of muscle or tendon.

Dr. Stromeyer's preface is succeeded by a long, we might say lengthy, introduction, wherein are discussed various abnormal conditions of the muscular system, with their bearings upon coincident deviations from natural conformation in the bony fabric, and the causes and treatment of the same. From among these observations we propose to select a few of such as possess most interest or bear more particularly on our subject. The author next lays before us a history of what has been done in times past for the advancement of this branch of our art; and subsequently proceeds to the successive consideration of deformities resulting from muscular contraction, of the feet, knee, and hip-joints, of the fingers and elbow, and lastly of the neck. Dr. Little's work is also preceded by an extended introduction, which includes the morbid anatomy and history of talipes, in the former part of which department he presents us with a great deal of matter possessing both novelty and interest. In the order we shall pursue in our subsequent analysis of the various materials we have collected, we shall endeavour, by a careful selection, synthetically to arrange that which, we trust, will form a useful though a brief compendium of the various opinions and modes of practice adopted by different operators.

Our subject offers a striking illustration of the essential connexion between the extension of anatomical research and the advancement of surgery; for, in tracing the causes which have retarded the progress of the curative means employed for club-foot in particular, probably none have operated so powerfully as a neglect to examine anatomically the sources of distortion, and an undue appreciation of the abnormal relation of the bones in the adult foot. Besides this, however, as Zeis remarks, the groundless fear of tetanus, exfoliation, or non-union of tendon after section, aided in no trivial degree in throwing obstacles in the way of attempting a cure by operation. To a certain extent, the remark of Scarpa is justified by dissection of deformed feet, viz., that the appearances presented are not necessarily identical even in the same form of distortion; but, as Dr. Little justly observes, it is in the infantile and unused foot that we must seek for the true anatomy of these altered relations, and not in feet, the abnormal form of which has been further modified by external agents, such as "improperly-directed mechanical treatment and the act of walking." The following is Dr. Little's arrangement of opinions relating to the causes of these distortions.

"1. That the primitive formation of the bones is unnatural and incomplete: 2. That the bones, being originally perfectly formed, become injured and distorted by causes independent of the formative process; viz. by pressure occasioned by the fœtus drawing the limbs into unnatural positions; by an improper situation of the fœtus in the uterus; or by certain ligaments becoming elongated, and the articulations distorted from contraction of some of the muscles and relaxation of others. 3. That, whatever may have been the condition of the bones on the occurrence of distortion, the act of walking displaces and injures them." (p. 22.)

Glisson, Camper, and Blumenbach, were amongst those who advocated the first of these opinions; but Scarpa, in a memoir, in which he described the most ordinary appearance on dissection, and the mechanical treatment of these distortions, remarked that the deformity of the individual bones was slight, and also noticed the important point, that the relation of the astragalus to the articular surfaces of the tibia and fibula was comparatively but little disturbed. The dissections of Dr. Little have induced him to confirm this statement of Scarpa.

"In every instance I have examined, either of an infant or adult, however great may have been the state of extension and adduction of the foot, some portion of each of the three articular surfaces of the trochlea of the astragalus was in contact with an equal proportion of the three articular surfaces presented by the tibia and fibula in the ankle-joint. The astragalus usually inclined inwardly, but only to such an extent as to cause the external surface of its trochlea to project from the ankle-joint somewhat further than the internal surface." (*Ib.* p. 29.)

The dissections of Jörg, Colles, Cruveilhier, disposed them to attribute the deformity in most instances to malformation of bone, which they considered as a serious, if not an insuperable, obstacle to the replacemen or reduction of the foot to its normal condition. Such was likewise the notion of Delpech when he first published upon the subject;* but he was subsequently induced to subscribe to the opinion that muscular action is the cause of the distortion. After expressing his own opinion, which we have quoted, Dr. Little concludes his summary of the investigations into the anatomical changes of varus, in the words of Scarpa: "none of the tarsal bones are actually dislocated; but in addition to the state of extension of the ankle-joint, they undergo rotation on their axes,

* Chirurgie Clinique, p. 166. 1823.

and the astragalus suffers less alteration of position than either of the tarsal bones."

In investigating the causes of these changes, we find the enquiry naturally resolves itself into two clauses, the source of mischief in the congenital distortion, and that which occurs in after-life. Club-foot and the analogous distortion of the hand are of far more frequent occurrence, as congenital malformations, than the production of any exciting cause which is brought into operation subsequently, and therefore quite independently of the fœtal period of life. This fact has induced early writers upon the subject, at a period when, as we have already observed, the changes in anatomical relation had not been accurately investigated, to refer these congenital distortions to an improper position of the fœtus in the uterus, or an undue and unequal pressure of this organ upon its contents. We are indebted to Rudolphi* for directing attention to the nervous centres as the probable seat of primary disturbance in most instances; an opinion which he founds upon the violent convulsive motions to which children are sometimes subject during uterine life, and also upon the early period at which these malformations are occasionally found, before the uterus can be supposed to have acted mechanically upon the embryo. Dr. Little refers to several embryos of from three to five months old, affected with talipes, which were collected by Rudolphi and his distinguished successor Müller, and deposited in the Berlin museum: some, he remarks, "exhibiting malformations or deficiency of the cerebrum and medulla spinalis; some anencephalous and others hemicephalous, both the hands and feet being affected in this manner, showing the extensive participation of the system in the disturbance and destruction of the nervous centres." (p. 33.) The frequent occurrence of coexisting talipes in both limbs has been cited by Cruveilhier as an argument in favour of his theory that malformed astragalus is the most frequent cause of the distortion. This opinion Dr. Little combats; and we fully coincide with him in regarding the above fact as rather favouring the inference that one common disturbing cause, such as is met with in a disordered nervous centre, must be sought for; a supposition which is by no means impaired by the occasional accompaniment of monstrosities in other organs. The hypothesis of Von Walther† is more curious than probable, viz., that "talipes is a natural grade of the development of the foot, and embryos of three or four months very frequently retain one or both feet in this state." The observation with which Dr. Little dismisses this theory is sufficiently conclusive. "From this (he says) it would follow, that every fœtus, at an early period of development, has two club-feet. But if talipes varus be a state of natural embryo-development, the enquiry may be instituted as to which of its natural stages talipes equinus and talipes valgus are respectively referrible. By whatever further stretch of imagination this theory may be applied to the explanation of talipes equinus, its application to talipes valgus is decidedly inadmissible."

A memoir, which had for its object the elucidation of the above question, (viz. the cause of congenital club-foot,) was read before the

* Physiologie, p. 319. † System der Chirurgie. Band i. p. 349. Berlin, 1833.

Academy of Medicine in Paris by M. Guerin, on December 11th, 1838; as it presents, probably, the most rational account of the matter, we will quote the leading points given in the Gazette Médicale for the same month.

"M. Jules Guerin draws attention to his idea that convulsive retraction of the muscles is the cause of congenital club-foot. He asserts that a great number of observations made on monsters and on the normal fœtus have proved that a relation exists between congenital muscular retraction and congenital alterations of the cerebro-spinal system, from the complete destruction of the brain and spinal marrow to the lesion of a circumscribed point of one of these centres. When club-foot exists together with other deformities of joints, they are all the result of convulsive retraction of the muscles, characterized by an extreme shortening of the greater part of the muscles of the trunk and extremities. When the retraction is limited to the muscles of the leg alone, forming club-foot, it may be referred either to a general convulsive affection which leaves unequivocal marks of its existence in the features of the countenance, the conformation of the skull, the direction of the eyes, the inequality of strength on the two sides of the body; or to a mere local lesion confined to certain nervous branches, and consequently to certain muscles which are alone affected. The convulsive retraction of the muscles is a complex action made up of three elements; the immediate shortening of the muscle, a certain degree of paralysis, and a consecutive arrest of development, which prevents its following, *pari passu*, the growth of the skeleton, and thus increases the primitive shortening of the retracted muscles. M. Guerin does not admit the existence of any other causes of club-foot, but allows that the compression of the uterus may produce a degree of deformity, which has not, however, the anatomical characters peculiar to club-foot."

M. Duval coincides with M. Guerin in these conclusions, rejecting, as a cause of club-foot, the pressure exercised on the fœtus by the walls of the uterus, in cases where the liquor amnii is deficient in quantity; and in this opinion he is joined by Breschet: but Cruveilhier maintains that in such cases the pressure of the uterus is an efficient cause of club-foot; and in proof of this he alleges that the foot, which is placed anteriorly, and is consequently more exposed to pressure, is generally affected, and, when both are deformed, the anterior one is more so. We have already noticed that Delpech abandoned his original notion that bony deformity was the cause of club-foot; it may be interesting to add that his reason for so doing was founded upon witnessing two cases of this distortion, of which one arose from the division of the popliteal nerve, and the other was consecutive to an abscess in the thigh with necrosis of the femur. In these, as in most if not all cases, the immediate cause is an inequality in the power of antagonist muscles, producing a deviation from the natural position in the direction of the contraction. We will conclude our remarks on this division of our subject, by quoting some excellent observations of M. Duval, which bear upon the point at issue. Having stated that a "bad position or an exaggeration of the natural position of the feet in the uterus" (not simply uterine pressure), "during some period of pregnancy, aided by a partial delay in development," may be an occasional though a comparatively rare source of the mischief, he proceeds as follows:

"A more frequent cause of club-foot is *a lesion of the cerebro-spinal system.* Infants born with paralysis of one or both sides are frequently affected with club-foot on the paralyzed side. The paralysis may disappear after a few months and the deformity remain; or it may persist, and in that case the deformed limb will generally become atrophied; indeed, at birth it is generally smaller and possesses less warmth than the opposite one, and this symptom will often enable us to decide if the deviation has arisen from disease of the nervous system, which may occur to the fœtus in the womb as well as to the infant possessing an independent existence. Arrest of development is by some persons, and particularly by M. Breschet, placed among the causes of *strephopody*,* and in corroboration of this idea it is stated that in one case the affected limb was three inches shorter than the sound one; in another there was a deficiency of five inches; in another case the last phalanges of the toes were wanting. Eight out of ten cases of consecutive club-foot arise from inflammation of the cerebro-spinal system, and are preceded by convulsions, paralysis, or muscular contractions. Why may not the same be the cause of the congenital disease? When a limb is first affected with paralysis either partial or complete, there is first retraction rather than actual shortening of the muscles; for a long time they may be brought back to and maintained in their normal length by means of a machine; but when the muscles are actually shortened, division of the tendons is necessary to restore the natural length and position of the limb." (*Revue Médicale.*)

Both Zeis and Bouvier present us with notices of the successive changes which tendons undergo in the process of reunion, (*Zeis*, p. 548; *Bouvier*, p. 437.) The former author quotes the experiments performed by Von Ammon upon horses and dogs; and although the results obtained from their several observations disagree in some points, (owing, probably, to the different class of animals selected for experiment,) they all coincide in leading to the important conclusion that union is equally solid in the end, even after the separation of the divided extremities has been very considerable; that, in other words, a wide separation is no obstacle to perfect reunion, although the process is, in such case, necessarily more protracted. We had an opportunity, a short time since, of examining a tendon (the tendo achillis), in which reunion was perfected subsequent to division by the knife; after short maceration, a longitudinal section presented a cartilaginous texture with distinct, glistening, tendinous fibres interspersed, and taking a parallel course from the normal portion above to that below, indicating a renewed continuity of the original structure.

We have already indicated the propriety of making the condition of an articulation the first object of careful examination, before having recourse to the knife for the relief of a contracted muscle; supposing, then, that such investigation prove satisfactory, it becomes a matter of much importance to discover the original source of mischief, the cause of the contraction, (which, as we have shown, may or may not be local,) and to employ such palliative or curative remedies as may appear adapted to the peculiarities of the case; for, be it remembered, although the section of a muscle or tendon be but a simple operation, yet it is by no means calculated to supersede, in every instance, all other remedies. Even Dr. Stromeyer appears willing to admit thus much, for he devotes some little space to the consideration of various palliative means; and as they are not likely to receive more commendation than they deserve at his hands, we cannot, perhaps, do better than select a few of his observations upon the subject.

"Every species of muscular contraction," he remarks, "whether the consequence of habitual spasm, or of irritation of the peripheral extremities of the nerves, or the nervous centres themselves, agree in regard to the result, that the muscular fibres are so deprived of their healthy tone, that neither antagonist muscles nor external force have the power to restore them to their natural length. . . . . Nature seems to

* From στρέφω, *to turn*, and πους, *a foot*, club-foot, so called by M. Duval.

hold out but little prospect of unaided reparation in such conditions, save, perhaps, in the spontaneous cure by anchylosis, as in white swelling, &c. But art has done a vast deal for these cases; and the most eligible and universally applicable remedy for contractions is to be met with in warm bathing, by the benefit derived from which many thousands have annually restored to them the use of their limbs." (p. 12.)

Dr. Stromeyer then goes on to observe that "friction may be a most valuable agent when appropriate cases are selected for its employment." In expressing his opinion upon the nature of the embrocation, or whatever form the application may be made to assume, he deems the sedative class as alone applicable to contracted limbs, whilst the opposite are indicated where the muscles have lost their tone or become paralyzed. In the former class, irritating liniments, electricity, moxas, &c. may be all employed in vain to relieve a condition which the simple section of the affected tendon or muscle has at once cured. Again, with respect to manipulation (shampooing), Dr. Stromeyer thinks that gentle friction with the hand, and the warmth thereby produced, may aid collaterally in the cure by restoring tone to relaxed muscle: but his experience has induced him to place but little reliance on the employment of antispasmodics or narcotics, which, whether exhibited internally or applied as embrocations to the surface, act, as he justly observes, more readily on the nervous centres than locally. Where deformity is solely the result of *local* irritation, leaving behind it a retracted condition of a certain set of muscles; the same author remarks that bandages and other mechanical apparatus may exercise a beneficial effect in some instances, but adds that the section of such contracted tendons is ordinarily the more ready and satisfactory means of completely removing the defect.

Supposing, then, that an operation is decided upon, it by no means follows that we are, therefore, to dispense altogether with the above-mentioned remedies; on the contrary, it behoves us to call in aid all such means as may collaterally assist in procuring a successful result from the use of the knife; and, as every prudent surgeon accustoms his pauper-patient to the air, diet, and confinement of the hospital before he proceeds to the performance of any important operation, so may bathing, rest, and the use of the apparatus to be afterwards applied, be advantageously employed for some little time before the section of a tendon: the counteracting tendency offered by opposing elastic or contractile tissues is thus decreased, and the limb is habituated to the irksomeness of confinement and compression. Previously to describing the operation, Dr. Stromeyer makes some practical remarks of a general kind respecting the incision, the knife to be employed, &c. That the external opening should, if practicable, be single and small, is an axiom to which all operators willingly subscribe; although, doubtless, it may be requisite occasionally to deviate from this rule where a large muscle is the object of division. A narrow knife is, however, under any circumstances, the most desirable, and in form it should be slightly curved, as a bistoury, but without any button-point, an addition which renders the section more troublesome, and by contusion of the neighbouring cellular membrane, is likely to give rise to consequences which may retard the healing of the external wound. Dr. Stromeyer has from this cause seen a tedious suppuration ensue. Mr. Whipple, of Plymouth, lays some stress upon the advantages derivable from an oblique division of the tendon, the reasons for which he thus states: "first, by so doing, you have a larger surface for nature to carry on her operations; secondly, you have the obliquely divided tendon in nearer approximation, and thereby secure a firmer ligamentous band than in the transverse division; and thirdly, the application of the instrument does not separate the lips of the wound, a desirable point, as the sooner it heals, so as to prevent the escape of lymph, the better."* Respecting the period of commencing the after-extension, Bouvier and Stromeyer are at issue: the former advocating the *immediate* employment of mechanical means for this purpose, whilst the latter deprecates what he styles the "so-called improvement" of our French author, by reason of the evils which he enumerates as resulting from this practice, of which the principal is the production of an unnecessary quantity of new intervening material, having the very prejudicial effect of impeding, if not of permanently preventing a complete restoration of the muscles to a state of healthy tone. In this point Dr. Little coincides with Stromeyer, whilst Mr. Whipple informs us that he has treated his cases, like Bouvier, by immediate extension. Dr. Zeis states that both he and Von Ammon have practised extension immediately after section of a tendon with good results, and therefore thinks it unnecessary to defer the application of the requisite apparatus. Thus there are a variety of opinions upon this simple point; for ourselves, although we think that in the long run the ultimate success may be nearly the same in each plan of proceeding, we are disposed to regard Stromeyer's as the more eligible, because less abruptly coercive, mode of treatment. The apparatus which is needed for the purpose of making and keeping up extension after division of the tendo achillis may be very simple; and as every surgeon would probably have his own contrivance for producing the required end, it is needless to describe any particular form. One positive and one negative property it is most essential to combine in the construction of the said machine, viz., the capability of graduating and preserving a steady extension, without making such local pressure as may induce injury to the superficial soft parts. For the purpose of avoiding this evil, the surgeon cannot be too careful in the frequent inspection of the apparatus, and in making enquiries of his patient whether pressure is felt more at one part than at another: we had occasion once to witness a very severe excoriation and partial destruction of the cutis on the instep from this cause; the apparatus was necessarily laid aside, but the case had fortunately proceeded sufficiently far for the cure to be completed without its readjustment. We need scarcely add, that it is by no means less important to the favorable issue of an operation, that the extending medium should not be removed or even relaxed, except under urgent circumstances, such as that above named, which leave no alternative. After perfect union has taken place, Dr. Stromeyer recommends that friction with stimulating lotions should be combined with gentle use of the limb. We are, however, anticipating our subject and outstripping our authors, and must therefore commence, *de novo*, with what we can collect of the *history* of tenotomy or tendon-cutting.

Of all the operations of this class, the cases in which the tendon

* For Mr. Whipple's paper and cases, we refer our readers to vol. xx. of the Med. Gazette, p. 826, and to an abstract of the same in our Fifth Vol. p. 285.

common to the two gastrocnemii muscles has been divided have far exceeded all others put together, both numerically and in the interest which that particular operation has excited in the profession; the deformities, for the cure of which it has been practised, being of frequent occurrence as congenital conditions, and likewise as the result of disease or accident. From the notice which Hippocrates takes of the subject of distorted feet,* we have little reason to doubt that he understood and successfully treated these deviations from a normal condition by simple mechanical means, such as bandaging and support of various kinds afforded; and, as Dr. Little remarks in the introduction to his work (p.xlvii.), we may even question whether he had not contemplated the practicability of some operation if he did not try one; for, after speaking favorably of the bandaging system, he adds, "atque quidem est curatio, et neque sectione, neque ustione, neque aliâ varietate quicquam opus habet." Be that, however, as it may, we read of no advance being made in the treatment of these deformities, until quite the latter end of the last century; it was then that the first recorded case of section of the tendo achillis was published by Thilenius,† who practised his profession near Frankfort. The subject was a girl, aged 17, who had been afflicted from a very early age with inverted club-foot on the left side, which caused her to walk on the outer border of the member; other means of remedying the deformity having failed, the operation was performed by cutting across the tendon, and thus dividing both it and the superjacent integument. The operator (by name Lorenz) having reduced the foot to its normal form, retained it in that position by means of a suitable bandage; and in six weeks reunion was complete, and the perfect restoration of the foot was ultimately accomplished. In 1811, Michaëlis published a memoir in which he described a modification of this operation, consisting of a partial division of the same tendon, by which a more ready extension of the remaining portion was admitted of, and, from the cases he relates,‡ was very successful. Michaëlis also operated on the flexor tendons of the leg and fingers; but as he considered immediate extension requisite for the cure of the case,—and the healing of his wounds was generally protracted,—Dr. Little's suggestion is not improbable, viz., that by the force he employed to place the limb in a proper position, the remaining undivided fibres were ruptured. The case related about the same time by Sartorius§ (of the Duchy of Nassau) was anything but encouraging, and his mode of proceeding certainly most barbarous. He exposed the tendo achillis for a considerable distance, and after freely dividing it, flexed the foot on the leg with such violence as to produce a loud cracking sound, doubtless ligamentous rupture at the least: the cure was tedious and eventually accompanied by an anchylosed articulation. Up to this date no attempt seems to have been made to avoid the extensive lesion of superficial soft parts, which different operators had deemed it requisite to make as a preliminary to dividing the tendon. Delpech made the first step towards attaining this desirable object, but his operation was far from complete;

* Liber de Articulis.

† Med. und Chir. Bemerkungen. Frankfurt, 1789.

‡ Hufeland's Journal. Band 33.

§ Siebold's Sammlung Chir. Beobachtungen, 1812.

and in consequence of the difficulties attendant upon the cure, he never repeated it. The case related by him* occurred five years after the publication of Michaëlis's memoir. The patient was between six and seven years old, suffering from simple talipes equinus: the first step of the operation consisted in passing a scalpel through both surfaces of integument covering the interspace between the tendo achillis and deep flexor muscles; a bistoury was then substituted, by which the section of the tendon was made from before backwards. A further modification in his plan consisted in allowing union to take place before extension was commenced; indeed for this purpose he maintained as much as possible the approximation of the divided extremities by an appropriate apparatus; and he further recommended that after the extension was completed, the improved position should be preserved until the newly-deposited material had become sufficiently consolidated to afford the requisite support in walking. M. Bouvier† tells us that he saw the subject of this operation at Paris, twenty years afterwards, and testifies to the completeness of the cure. This brings us down to the time of Stromeyer, who, in spite of the early want of support and the many severe criticisms on his reintroduction of this operation, has completely succeeded in establishing it as one of the most useful as well as certain improvements in modern surgery. We have already adverted to the chief peculiarities which characterize Dr. Stromeyer's general mode of operation, viz., the *small* and usually *single* puncture of the skin, the *complete* section of the tendon, and the *delay* in making extension, and have noticed the palliative and mechanical treatment applicable to some cases; we will now take up our subject more in detail, and consider severally and in order, the operative treatment of deformities of the *feet*, *knee* and *hip-joints*, *fingers*, *elbow*, and *neck*.

It is much to be regretted that the profession does not more readily fall into the adoption and employment of a conventional nomenclature, without constantly seeking to improve upon that which exists. It rarely happens that a fresh labourer enters upon any particular subject, whether new or old, without restlessly seeking to impress an extra stamp of novelty upon the results of his investigations, by calling old things by new names, forgetting in this, as in matters of far higher importance and of wider interest, that alterations are not necessarily improvements. We do not, however, consider this remark as applicable to Dr. Little, whose nomenclature we are willing to adopt. Our own compound word "club-foot" has always been, and always may continue to be, in common language, applied generically to all three forms of distortion, viz., the inversion, eversion, and the simple elevation of the heel. Synonymously with this term Dr. Little proposes employing the classical word 'talipes' (hitherto applied to one species only) as a generic term to include all those deformities of the feet produced by contraction of certain muscles, to restrict it to deformities from this source, and to use the terms *varus*, *valgus*, and *equinus* to designate the specific forms of these diseases. The least complex of the deformities alluded to (which are thus classified under three heads) is the *talipes equinus*. This form consists in a simple extension of the foot, by which the heel is elevated, and the subject of

* Chirurgie Clinique de Montpellier. Tom. i. † Mémoire, p. 427.

the affection rests and walks upon the phalanges, or rather the heads of the metatarsal bones; an angle is thus formed between the phalanges and metatarsus, which varies according to the perpendicularity of the latter, and posterior to which no portion of the plantar surface touches the ground: in the most aggravated cases, the angle is rarely produced beyond a right angle. In this, as indeed in all the forms, the habitual disuse of the limb impedes the development of both hard and soft parts; the bones are usually shorter and more slight than on the healthy side, and the muscles are small and flaccid; and Dr. Little remarks that the whole vitality of the limb is below par. Although the preceding is the least complicated form, the *talipes varus* is the most frequent of occurrence. This variety combines extension with adduction of the foot; and to these two characteristics a third may be added, viz., "a rotation of the foot somewhat analogous to supination of the hand taking place to a greater or less extent, according to the severity of the disease;" the inner edge of the foot is thus raised from the ground, forcing the sufferer to walk on the outer margin only. In the *talipes valgus*, which is comparatively rare, the distortion is of an opposite character; we describe it in Dr. Little's words:

"It may be regarded as the opposite state to talipes varus, and like it consists of a threefold alteration of the position of the foot, there being partial bending of the ankle, *ab*duction, and a rotation of the foot; but this rotation foot takes place in the opposite direction to that in talipes varus, as in talipes valgus, the external *edge* of the foot is removed from the ground. The rotation in a complete case of talipes valgus is so great that the patient, in the act of walking, does not touch the ground with any part of the sole of the foot, but treads entirely upon the inside of the instep, and upon the malleolus internus. In short, the sole of the foot is directed completely outwards and a little backwards, the ankle is held in a state of semiflexion, the anterior half of the foot (the metatarsus and toes) not touching the ground." (p. 4.)

Although each of these forms frequently exists independently, the *degree* of adduction produces modifications of various kinds between the talipes equinus and varus.

We have already noticed the alteration in bony configuration accompanying these deformities; we shall now make some enquiry as to the muscles which are implicated in the disturbance of the normal relation of the parts affected. This is a point of great practical importance, one indeed on which the successful treatment of the case essentially hinges. With regard to the simple extension of the foot constituting talipes equinus, all our authors agree in referring the elevation of the heel, as one might readily predicate, to a permanent approximation of the origin and insertion of the gastrocnemii muscles. This shortening may, however, arise from various causes; it may be the result of continued tonic spasms, referrible either to lesion of a nervous centre, or to functional derangement of some important organ,* or it may be the consequence of actual loss of structure, as from a large sloughing ulcer, &c.,† or again, the soft parts may contract unnatural adhesions, or the muscles become permanently shortened from the restraint of a protracted unnatural position, or from

* The reader may consult, inthe Appendix to Dr. Little's work, cases ix. xxvii. xxviii. xxxi. This author states that he has treated one case of non-congenital talipes which was distinctly referrible to the irritation accompanying teething.

† Id. cases x. and xi.

paralysis of their antagonists. We have indicated in an earlier part of this article that some of these cases may be benefited by a palliative mode of treatment, but all may, and frequently do, require the knife. As section of the tendo achillis alone is required for the restoration of the foot, in most cases of talipes equinus, we may here notice the mode adopted to effect that object by Stromeyer and Little. Having selected "a small, curved, sharp-pointed bistoury, with a concave edge, the cutting part of the blade seven tenths of an inch in length, and the greatest width one tenth of an inch," in order that the external puncture may be as small as practicable, the surgeon, according to Dr. Little, proceeds as follows:

"The patient being seated, an assistant supports the knee, whilst another, drawing downwards the patient's heel with his left hand, and pressing upwards the toes and front of the foot with his right, produces the necessary tension in the tendon proposed to be divided. The operator, after feeling the outline of the tendon with the left fore-finger and thumb, passes the bistoury through the skin, one or two finger's breadth above the malleolus internus, with one of its sides turned towards the tendon, and the other directed towards the deeper muscles and the tibial vessels and nerves. On being satisfied that the point of the knife has been passed beyond the external edge of the tendon, and has nearly reached the skin of the opposite side, the knife is turned so as to bring the cutting-edge to press against the anterior surface of the tendon, which is then divided by the action of withdrawing the knife from the limb, and commonly, by a single stroke. The complete division of the tendon is known by the immediate cessation of the tense resistance, by hearing a distinct snap, and by feeling, before the knife is wholly withdrawn, that nothing remains undivided except the flaccid integuments. The operation does not occupy a quarter of a minute, and is almost bloodless, as usually not more than a single drop of blood is effused." (p. 30.)

The small size of the wound is particularly and justly insisted upon by all our authors, as an essential and most important improvement upon the earlier mode of operating; and we believe that the want of success of all the operators, down to the time of Delpech inclusive, was attributable to the large extent of external wound, which they considered a requisite part of the operation. In confirmation of this position, Dr. Little states that "he has operated on seventy-three cases of contractures of the ankle and knee-joints, treated by division of various tendons; amongst which there was no instance of the puncture not having immediately united by adhesion." This result he ascribes to the smallness of the wound, and "the non-disturbance of the healing process by precipitate attempts to straighten the limb." (p. 29.) The wound, then, should be allowed to close before extension is commenced; for this purpose two or three days are generally sufficient, during which interval the limb should be laid on its outer side, on a pasteboard splint; and even after that Dr. Stromeyer recommends that the obstacles offered by resisting ligaments and fasciæ should be very gradually conquered, as their shortened condition is no trivial additional impediment to the reduction of the foot. Two apparently trifling but really valuable hints of Dr. Little, in relation to the after mechanical treatment, are worth citing; the one is, "that the skin should be protected from friction and uneven pressure by the application of an elastic cotton-web bandage, and by filling up with wadding those inequalities of the surface of the tarsus which arise, in many instances, from unequal projection of the bones;"

the other is, where the foot is very uneven and tender, "to place an air-cushion between the sole of the foot and the foot-piece of the apparatus, in order to produce an equal distribution of the pressure over the whole sole." It is requisite to continue the use of the extending apparatus, even after the cure is effected, to obviate the natural tendency to contraction.

Many of the foregoing remarks are equally applicable to the two other forms of talipes, more particularly the varus, many cases of which require the same treatment as the equinus, viz. simple division of the tendo achillis; whereas, on the other hand, Dr. Little states that in some of the more obstinate cases of pure talipes equinus he has "found it necessary likewise to divide the tendons of the tibialis posticus and flexor longus pollicis muscles:" of this exception his cases vii. and viii. afford illustrations. The treatment of talipes varus, therefore, consists, as in the form equinus, in the division of the tendons of the opposing muscles, which may be either limited to the gastrocnemii, or may extend, in some of the severer and longer-standing cases, to section of the tendons of the tibialis anticus and posticus, with the extensor and flexor proprius pollicis;† for exemplifications of these the reader may refer to the appendix of Dr. Little's work.* In several of these cases, which are characterized by marked inversion of the foot, the dependence of the deformity in great measure on contraction of the tibialis posticus has been indicated by Dr. Stromeyer; he has, therefore, frequently divided this tendon, and in one case (viii.) this only; although he admits that his more recent experience inclines him to believe that as much benefit may be obtained for the relief of this distortion by the proper application of mechanical means alone as by the operation; and this opinion is confirmed by some of his cases, and his own summing up and inferences at p. 81. In one case (vii.) it was found requisite likewise to divide the plantar aponeurosis.

That muscles which are naturally antagonists of one another occasionally require division in one and the same case, may be better comprehended by reflecting on the altered bearing of their action, resulting from their abnormal course in cases where the deformity is of an aggravated kind. This remark is peculiarly applicable to the muscles of the anterior tibial region, and is referred to by Dr. Little, and delineated after one of his dissections. (p. 12.) For an account of the best mode of dividing the muscles last alluded to, we again turn to the description given by Dr. Little.

"The division of the tendon of the posterior tibial muscle is in my opinion best accomplished at the distance of two or three fingers' breadth above and behind the internal malleolus. The point of a strong and straight bistoury should be introduced through the skin at the outer edge of the tendon, and passed between it and the tendon of the long flexor of the great toe, directed towards the tibia. As soon as the knife reaches the bone, the handle should be depressed outwardly, and the point carried internally beneath the posterior tibial tendon, and continued outwards, until the surgeon is satisfied that the point has passed beyond the inner edge of the tendon. He may then feel that he has the tendon upon the edge of the knife, when, by a few slight cutting motions, he may divide it without difficulty. No snapping sound, similar to that which follows the division of the tendo achillis, is heard, when the

* Cases xiv. xviii. xxiii. and xxiv., and the remarks appended to case xvii.

section of the posterior tibial tendon is accomplished; as the fleshy fibres of this muscle take their origin so low towards the malleolus internus, that they prevent the occurrence of any considerable retraction of the superior end of the tendon. The most favorable situations for dividing the tendons of the tibialis anticus and flexor longus pollicis muscles are where the former passes in front of the ankle-joint, and where the latter is felt most prominently in the sole of the foot, in those cases where division is required. . . . . The division should be made from within outwards, in order not to endanger the neighbouring structures. The recoil of these muscles, on their tendons being divided, is distinctly felt and heard." (p. 31.)

In the third form, *talipes valgus*, Dr. Little's experience induces him to refer the deformity chiefly to the obstruction offered by the peronei muscles; it is likewise usually requisite to divide also the tendo achillis (as exemplified in Dr. Little's case xxviii.), and even, strange as it may seem, the tendon of the tibialis anticus, before the foot can be restored to its natural position. The comparative rarity of these cases is evidenced by the fact that Dr. Little has witnessed but two of this form of distortion, whilst he has had presented to him more than one hundred instances of the other two species. The section of the peronei tendons may be effected in the same position, and (where both are the subject of operation) even by the same punctured opening through which the tendo achillis is divided. Dr. Stromeyer states that he has treated this form by section of the tendo achillis only, as in case xxii., related in his work; and again, the succeeding case is an exemplification of the successful employment, at a very early age (three months), of mechanical means without operation, for the cure of talipes valgus: the apparatus consisted of a small boot, with a long spring, adapted so as to operate in a direction opposed to the abnormal eversion of the foot; the cure was completed in a few months.

The notice of this case leads us to remark upon the *age* at which it may be deemed advisable or most desirable to employ the operative treatment in cases of club-foot. The youngest patient amongst Dr. Stromeyer's was a boy of eight months old, in whom the tendo achillis was successfully divided for talipes varus.* A case of spasmodic congenital varus was likewise cured by Dr. Little, in a child, aged twenty months, by section of the same tendon.† Of Mr. Whipple's nine cases,‡ the youngest was fourteen months old. With regard to the other extreme, we may presume that a cure is practicable at any period of life; for Dr. Little narrates a case (xxxiv. p. 258) of "non-congenital distortion from contraction of the gastrocnemii and other muscles on the posterior aspect of the right leg, converted by constant exercise into a deformity resembling, in external appearance, talipes varus, originating from paralysis: cured by division of the tendo achillis, and subsequent mechanical extension, after the distortion had existed *forty-eight* years." The subject of the operation was in his fiftieth year. A similar case is related by Dr. Stromeyer (p. 82), of a female whose deformity had existed since her fourth year, and who was operated on at fifty; but her habits had been comparatively sedentary, and she had employed crutches, by which an aggravation of the distortion was, to a certain extent, prevented. Dr. Little's case is, therefore, the more remarkable of the two. We had almost omitted to observe that this latter gentleman

* Case xi. p. 81. † Case xvi. p. 159. ‡ Noticed in our Fifth Vol. p. 285.

notices a fourth form of talipes, in the production of which the tibialis anticus and extensors of the toes are the active agents. This deformity consists in abnormal *flexion* of the foot, the heel alone touching the ground; and it is therefore opposed to talipes equinus, bearing the same relation to the talipes valgus as the talipes varus and equinus respectively hold to each other. Dr. Little has treated a case of this form by simple extension, but thinks that the tibialis anticus or extensor communis may, in aggravated cases, require division. He has named the deformity *talipes calcaneus.**

In closing the subject of deformities of the feet, Dr. Stromeyer devotes a brief chapter to the notice of what he terms "Plattfuss," a word with which our *duck* or *splay-foot* to a certain extent corresponds. This affection, when aggravated in form, becomes both troublesome and painful, and therefore deserves the attention of the surgeon; yet but little heed appears to have been paid either to its pathology or treatment. Dr. Stromeyer remarks that the condition of splay-foot appears clearly to depend upon "atony of the plantar aponeurosis, and the ligaments which bind the different bones to one another, and connect them to the tibia and fibula. "These tissues yield," he adds, "to the superincumbent weight, and thus not only is the arch of the foot annihilated, but the foot also gives way in an outward direction, because the ordinary action of the gastrocnemii and tibialis posticus muscles is so far interfered with by the relaxed and unsteady condition of the articulations, as to produce a drawing or pressing of the tibia inwards." A shrunken state of the limb is rather to be regarded as an effect than as a cause of the affection. Dr. Stromeyer relates four cases of its successful treatment; and, as they vary but little, we will select one as an example of the rest.

"H. K. ætat. 16, of lymphatic diathesis, whose employment had been for half a year previously in a merchant's office, presented himself with a splay-foot to Dr. Stromeyer, in December, 1831. This affection, which had existed for some months, was palpably the consequence of standing continually behind the counter, on a cold floor. He was in pain when either standing or walking, the foot was bent outwards, and the plantar arch quite destroyed; moreover, distinct fluctuation might be perceived between the bones of the tarsus, and a sensation of heat was communicated to the hand. Twelve leeches were applied, and the foot afterwards bathed with Goulard's lotion. In four days the heat and fluctuation were thus dispersed, and a blister was then applied to the inner border and part of the arch of the foot, and kept open. After the healing of the blister the foot was found to have regained its normal form. The patient was ordered to wear a bandage and lace-boot, by the use of which the cure was rendered complete." (p. 101.)

Dr. Stromeyer adds, that the aid of splints and spring-supports is occasionally desirable in the more obstinate forms of this complaint.

The next division of our subject relates to contractions of the knee-joint, and that which Dr. Stromeyer terms "false anchylosis" of this articulation. For the materials whence to compile a summary of the treatment of this affection, as well as of those which follow, we shall be principally indebted to Dr. Stromeyer's work, as both Dr. Little and M. Bouvier take leave of us here. Many of our preceding *general*

* This form of talipes is also noticed and described by Mr. Whipple, in his paper already referred to. See Med. Gazette, vol. xx. p. 826.

remarks may be equally applied to the subsequent complaints which we have to notice; therefore, we trust to keep our observations within the limit of tediousness, by allotting a comparatively brief space to the consideration of the individual sections which compose the remainder of our subject.

*Knee-joint* contractions are more frequently the result of disorganization consequent on chronic or long-standing inflammation and suppuration, than dependent, as in the instances we have already discussed, on a "dynamic influence," such as muscular spasm. Be that, however, as it may, the operation of dividing the hamstring muscles is neither less essential, nor has it proved less efficacious in the cure of this affection. These cases are sometimes complicated with contraction of the feet; and Dr. Stromeyer informs us that he has seen a "sort of chronic tetanus," affecting not only all the limbs, but even extending to the muscles of the face. In a case where both knees and ankles were implicated, M. Duval, of Paris, divided the tendons of the gastrocnemii and hamstring muscles on either side, and the result was satisfactory; but Dr. Stromeyer does not express himself as favorably disposed to this wholesale way of operating, but rather recommends the employment of palliative measures, such as perseverance in warm-bathing, &c. Cases xxviii., xxix. and xxx. of Dr. Stromeyer's book are exemplifications of the beneficial application of the operation to the radical cure of contracted knees. The first is termed "false anchylosis" of the knee, and occurred in a lad, twenty-one years of age, whose complaint originated in scrofulous inflammation of the joint four years previously. The articulation formed an angle of nearly 110 degrees, and was only slightly moveable by the employment of considerable force. An extending apparatus (delineated in Tab. v. fig. 1 of the work,) was employed advantageously up to a certain point; but an attempt to push its use further, produced so much local and constitutional disturbance, as rendered it necessary to give up the hope of obtaining any permanent benefit from its continuance. An operation was therefore had recourse to: the tendon of the biceps cruris was first divided, with the same sort of knife, and in the same simple way as section of the tendo achillis is performed; the extending apparatus was then reapplied, but it was found indispensable, at the end of a fortnight, to divide in like manner the semi-membranosus and semi-tendinosus muscles. After the lapse of three months the limb was perfectly straight. In case xxix., the nature and cause of which were similar to the former, it was found necessary to divide only the inner hamstring muscles, and the result was equally favorable. Case xxx. was somewhat more complicated, the contraction, at an acute angle, having succeeded scrofulous suppuration of the joint, which lasted for eight years. The fascia was, in this instance, likewise implicated as a cause of the abnormal position of the articulation; it was, therefore, found requisite to divide portions of it, more especially near to the intermuscular septa, as well as all the hamstring muscles; and this was effected at various operations, the apparatus being applied in the intervals between each: the treatment proved ultimately successful. The remaining cases, illustrative of these affections, were either benefited or cured without operation, as by mechanical extension, friction, warm bathing, &c. We may notice, by the way, that Dr. Little relates one case at the end of his work, to exemplify the operation of dividing the hamstring muscles; the instance was analogous to those we have cited, and its issue moderately successful.

Our space will not allow us to analyze the half-dozen pages which Dr. Stromeyer gives to the consideration of *hip-joint* disease and the resulting deformities, especially as it is a little foreign to the object of our present article; we must, however, find room for a brief sketch of one case, in which the pectineus and sartorius muscles were divided for contraction at this articulation. The patient, who was a girl of nine years of age, was placed under Dr. Stromeyer's care in September, 1837. A twelvemonth previously she had had measles, and going out earlier than was prudent, she was almost immediately attacked with disease of the hip-joint, accompanied by pain and contraction in the knee. Active antiphlogistic measures, to a certain extent, arrested the symptoms; but flexion from contracted muscles of the right hip-joint was ultimately so decided that the thigh touched the abdomen. Attempt at extension aggravated the pain; and as an apparatus had been for some time worn with very little improvement, it was discontinued, together with the other palliative measures, and an operation was decided on. We may premise the description of this, by observing that attempts to *extend* the limb clearly indicated that contraction of the pectineus and sartorius muscles, especially the former, was the obstacle that interfered and prevented it.

"On the 5th of March, 1838, the division of the pectineus muscle was effected. The patient was placed on a sofa, and whilst one assistant fixed the pelvis, a second extended the bent extremity, so as to put the pectineus on the stretch; the left index-finger being then placed, hook-fashion, behind the outer border of this muscle, about one inch and a half from its origin, a well-curved bistoury was made to pierce the skin and its upper half. This portion was then first divided beneath the integument, and a similar operation then performed on its under and inner half, so that only a few drops of blood escaped by the external aperture. To accomplish the section of the sartorius, the knee was adducted, so as to render the muscle tense; it was then seized between the finger and thumb, and divided beneath the skin at a distance of about two inches and a half from its origin. The extremities retracted about an inch. After the completion of both steps of this operation, the thigh was gently straightened and without any pain; an extending apparatus was then adjusted and worn for a fortnight, at the end of which time the limb exhibited no tendency to recontraction." (p. 120.)

This case was still under treatment when the report was made; but Dr. S. anticipated perfect restoration of the limb from careful exercise and warm bathing during the ensuing summer.

Dr. Stromeyer next informs us that he has relieved *permanent flexion of the fingers*, by removal of hardened and contracted cicatrix, or division of the flexor tendons. Case xlii. illustrates the successful adoption of the former treatment: the subject was a child, five years old, who had burnt the side of her hand; and the little finger was bound down to the palm by an indurated cicatrix; this portion was excised and the hand extended on a splint applied to the dorsum: the wound healed in ten days, but after a time the tendency to contract again declared itself, and it was necessary to resume the employment of the extending apparatus, which was continued at night for some time before the cure was completed. Of the latter form of cure (viz. by section of tendon), case xliv. is an example; this we shall quote, because we think it valuable as a precedent, the imitation of which may at any rate be attempted before a case is given up as incurable, or a finger sacrificed as not only useless but an incumbrance.

"A butcher, forty years of age, had, fifteen months previously, abscess of the fore-finger in the right hand, which required several openings: suppuration took place also on the dorsal aspect of the little finger. After two months treatment the parts healed, but the thumb, fore and little fingers, remained permanently flexed; and it was sufficiently evident, when extension was attempted, that the contraction of the flexor muscles was the source of resistance. The thumb was at once righted when its long flexor tendon was divided beneath the skin opposite to the first phalanx: but the superficial and deep flexor tendons of the index and little fingers being completely separated from one another, it was found necessary, after section of the former, to turn the knife towards the phalanx in order to divide the latter. After this double operation the fingers yielded to extension: the little finger was placed for a short time on a splint, and after employing passive motion for a fortnight, the patient was enabled to return to his business." (p. 126.)

Dr. Stromeyer notices also the permanent flexion of the little finger from contraction of the palmar fascia, a condition occasionally met with in individuals, such as those afflicted with club-foot for instance, who are accustomed to walk with and lean heavily on a stick; for the removal of this form of contraction, our author has found it requisite to divide integument as well as fascia. For the cure of *permanently flexed elbow-joint*, Dr. Stromeyer has never met with a case requiring division of the tendon of the biceps, an operation which is recorded as having been several times performed; he has, however, successfully treated many cases, which, he observes, are most commonly the consequence of inflammation of the articulation, by the employment of an extending apparatus, similar to that before alluded to as applicable to the knee-joint. Should section of the biceps cubiti tendon be desirable, the operation would, with the requisite precaution of avoiding the neighbouring artery and nerve, be simple and without hazard.

The affection denominated "*wry-neck*" is one of considerable physiological interest and practical importance; and the operation of dividing the sterno-mastoid muscle for its cure, is one of the earliest belonging to that class of operations of which we are now treating. As long since as 1670, Roonhuysen* divided this muscle together with the superjacent integument; and more recently the operation was performed by Tulpius and Sharp, the latter of whom gives a description of it in his "Treatise on the Operations of Surgery."† Boyer, Dupuytren, Chelius, Dieffenbach, Brodie, and many other surgeons, have adopted this radical means of cure successfully. It appears doubtful whether the modification of merely piercing the integument and dividing the muscle, in the same way as recommended for division of the tendo achillis, is due to Dupuytren or Dieffenbach; but be that as it may, it is doubtless the most desirable mode of operating, where it is practicable. The best spot to select for the section is, as Dr. Stromeyer remarks, "that which can be isolated with security, and that is at the tendinous portion near to the external extremity, where also the local retraction is comparatively trifling, as is the case in division of the gastrocnemii tendon; whereas section of the fleshy part of the muscle tends to the production of greater swelling about the divided extremities." (p. 129.) In severe cases,

* Gerardi Blasii, Observ. Med. rarior. Amstel. 1700. Pars 2.
† London. 1740. Chap. xxxv.

Dr. Stromeyer admits the propriety of commencing extension immediately after the operation, as the tendency to retraction is in some instances so great, that directly reunion has taken place, the abnormal flexion of the neck recurs; especially where the disease has been of long standing, and the energy of the constitution is much impaired. Mechanical extension and stimulating embrocations may then be serviceable. "A very singular circumstance," remarks Dr. Stromeyer, "and one which has not, within my knowledge, been heretofore noticed, is the coincidence of wry-neck with abnormal position of the fœtus in the uterus, so that either a buttock presentation results, or it is found requisite to turn in delivering." (p. 131.) Several of these cases have been made known to Dr. Stromeyer by his friends, some of which he afterwards relates, where wry-neck appeared to be the consequence of protracted and difficult labour, requiring the assistance of the forceps and considerable force for the extraction of the child. Dr. S. does not mean to ascribe the above condition to misplacement of the fœtus in all cases, but believes that, like other similar affections, it may result from inflammation, or be the consequence of primary irritation in the cervical portion of the medulla spinalis. The latter explanation would, indeed, appear to be the most rational mode of accounting for this sudden spasmodic contraction of a muscle which occasionally occurs in healthy adults, from an overstrained exertion, a condition to which other muscles are obnoxious besides the sterno-mastoid, as the biceps cubiti for example. Tonics, sea-bathing, &c. aid, to a certain extent, in relieving some forms of this painful complaint, for painful it sometimes is as well as troublesome; but both medicinal and mechanical treatment generally prove useless, except in cases where the affection is of recent date, and is distinctly traceable to functional disturbance of some important organ, such as the uterus; in support of which opinion we may cite the authority of Travers,* Brodie,† and others. The operation, although not quite so simple as the section of some of the tendons to which we have referred, is, nevertheless, neither difficult nor perilous. It may be performed, according to Dr. Stromeyer, in two ways, either from without inwards or from within outwards, (i. e. as regards the superficies of the muscle, not the median line of the body;) the former section is only to have the preference where the muscle stands boldly out in relief; it would otherwise be hazardous. We will cite an exemplification of each mode of operating, and for the former we select case xlv. The patient was a lad, eight years old, who had been for five years the subject of wry-neck, probably the result of indurated tonsils and the irritating treatment (locally) employed for their reduction. The sternal portion only of the right sterno-mastoid was shortened by one inch and a half. Mechanical extension had been fruitlessly employed for more than a year. The operation was thus accomplished:

"An assistant depressed the right shoulder and drew the head towards the opposite side, whereby the muscle was put strongly on the stretch. I now raised a fold of integument of a finger's breadth over the muscle at its attachment to the sternum, and pierced it at its base.‡ After two thirds of the blade had been made to perforate

* A further Enquiry concerning Constitutional Irritation, p. 283.

† On Local Diseases of the Nervous System.

‡ Reference is here made to an engraving representing the form of a knife employed, which was long and bistoury-shaped, but with the *convex* edge cutting.

the skin, the section was effected and readily recognized by the cracking noise which accompanied the separation of the divided ends; and the opposite corresponding muscle being now allowed full play, was immediately brought into action, and drew the head nearly straight. In this posture the head remained for a few seconds only and then resumed even a more bent position than before the operation. Three days subsequently the extending apparatus* was affixed, and the cure was completed in four weeks." (p. 133.)

The severer forms of wry-neck are not, however, solely dependent on or curable by division of the sterno-mastoid muscle; the clavicular portion of the trapezius is sometimes implicated, and may, therefore, also require to be divided, in order to effect a radical cure of the complaint. Such was the case in the instance we are about to cite in illustration of the other mode of operation, viz., from behind forwards. As the case is too long to quote verbatim, we select the more important passages only.

"Miss N. N., about thirty years of age, was affected with *habitual spasm* of the sterno-mastoid muscle, which had existed in a modified condition for some time, but had during the last two years assumed a more aggravated form. Having resisted all palliative treatment, the operation of dividing the sterno-mastoid was decided on. As it was supposed that the sternal portion alone was the seat of spasm, that part of the muscle was divided in the same manner as in the former case; but after the lapse of a month, it was deemed requisite to repeat the operation on the clavicular portion, which was accordingly done from behind forwards. A similar shaped knife was employed for this purpose, but with its *concave* edge cutting. The portion to be divided was seized between the finger and thumb of the left hand, and the blade was passed beneath the raised muscle, and the section accomplished in withdrawing the former, without further wound of the integument than the one point of perforation. Although the patient was much benefited by this second operation, a third was necessary to complete the cure; this was performed four months subsequently, and consisted in dividing in the same manner the anterior border of the trapezius muscle." (p. 137.)

Dr. Lehmann, of Torgan, has successfully treated a similar affection by section of the sterno-mastoid of the right side. The case in question was congenital, and had resisted the mechanical treatment. After section, the divided extremities separated for two fingers' breadth; the wound healed in two days, but considerable pain all along the course of the muscle lasted for three days; in twelve the patient was well.†

The remaining four cases which conclude Dr. Stromeyer's volume are related to illustrate the treatment of spasm-affected muscles by medicinal means. The first (case li.) was "spasm of the sterno-mastoid and scaleni muscles of one side;" the second, third, and fourth, (cases lii. liii. liv.,) "wry-neck, consequent on inflammation of the cervical vertebræ;" and lastly (case lv.), "retraction of the muscles of the neck following inflammation of both cervical and dorsal vertebræ." The first case was much relieved by large doses of stramonium and subsequently

* For a perfect comprehension of this somewhat elaborate and complicated apparatus we must refer our readers to the engraving in Tab. vii of Dr. Stromeyer's work. The patient is placed horizontally on a couch, and the body is fixed by a girth round the waist and straps extending thence to the bed-foot: the shoulders are also fixed. The head is then made to repose on the sound side, and a broad strap or collar is placed so as to catch the chin on the opposite side, whence proceed straps which pass backwards and are made fast, through the medium of a ring, to the extremity of a short, curved, metal rod, springing vertically from the head of the bed, so that its summit extends a little above the head of the patient.

† Berlin. Medicinisch Zeitung. No. 2. Jan. 1839.

of opium, but the patient ultimately died in great suffering, and the only morbid appearance that presented itself was abnormal firmness of the brain and spinal cord. The other cases yielded to the exhibition of mild mercurial doses internally, and the use of mercurial friction, with leeches and blisters: the last case also required the employment of a moxa.

Our subject is now nearly brought to a close; indeed, we have only to add, in a few words, what we trust to have already rendered almost superfluous, a summary of the relative merits of the works, the contents of which we have been analyzing. The order and arrangement of matter adopted by Drs. Stromeyer and Little are very similar, and certainly present many advantages to the reader who may wish to study his subject, without the interruption offered by extended illustrations of each form of disease and its appropriate treatment; we allude to the separation of the cases from the body of the treatise. Dr. Little is in most points a disciple and close follower of Dr. Stromeyer; the principal merit in the labours of the former consisting, as we have already noticed, in his careful and commendable researches into the anatomy of distorted feet. The drawings which are interspersed throughout Dr. Little's work, exhibiting the relative appearance of the various forms of talipes, both before and after operation, are cleverly executed, and form a very valuable addition to his treatise. We hope, however, that when Dr. Little publishes, as we trust and expect he will ere long, a more comprehensive second edition, he will see the advantage of giving his cases a little more concisely; we even think that his volume would not lose much of its practical value by the entire omission of some of the cases, the lengthened details of which unnecessarily increase its bulk. The majority of the cases are, however, interesting exemplifications of the principles laid down, and we therefore commend them, together with the remarks appended, to the careful perusal of our readers.

The greater portion of Stromeyer's cases, though even more numerous, are given more succinctly. As we have already seen, the work of our German author comprises a larger field, and is, therefore, more complete as a systematic treatise on the subject we have been reviewing.

Bouvier's memoir, which is limited to the discussion and treatment of distortions of the feet, presents but little, calling for any particular comment. His claims to improvement in the operation are trivial: we have shown that the utility of employing *immediate* extension, a principle which our French author strongly advocates, is at the least questionable; while his fear lest the intervening new deposit should, after a few days, become too solid and resisting to yield, is without foundation.

Before concluding, we must say one word in favour of Mr. Whipple, whose merits ought not to be forgotten in our review of the more extended researches and labours of others. The paper of this gentleman, read before the Medico-Chirurgical Society, as well as his subsequent communication to the Medical Gazette, containing the detail of several cases, exhibit, without any assumption of merit on his part, a practical knowledge of his subject, both in a pathological and surgical view, which, taken in conjunction with his unquestionable claim to priority in the introduction of the operation amongst us, entitle him to the thanks and acknowledgments of the profession generally.

